# Prof. Liautard of the American Veterinary College on the Subscription Plan

**Published:** 1884-07

**Authors:** 


*Prof. Liautard of the American Veterinary College on the Subscription Plan.*

*My dear Doctor:* I will finish my letter by a few remarks upon the Subscription Plan," which you may use in your JOURNAL if you so desire. In the year 1864 I had been established in New York for about four years, and commanded a practice of some $4,000 per year, the good will of which I turned over to the Trustees of the New York College of Veterinary Surgeons for the sum of $500, with the privilege of purchasing the ground, which I held on lease, at a low rate from the owner.

The Trustees of the N. Y. C. V. S. issued the "Subscription Plan," and I advised many of my friends, my own clients, to join it. I succeeded so well that the list of subscribers soon amounted to some fifty names. When the American Veterinary College was opened the same plan was adopted, and continued until about four years since, when, by degrees, it was slowly abolished; first by declining to accept any new subscribers, and again by not renewing subscriptions as they expired. Three years since this College only had about a dozen subscribers, and to-day we have none.

Now who has suffered by this plan? No one, I believe, but myself. Who benefited by it? The students and graduates who to-day find fault with it. Who was money out of pocket —who had to give time and labor to the school—but myself. Was I excusable? I leave it to the profession. Single-handed I have done this, and raised the clinical department of the College to its present condition, as well as established a free clinic. I never had either a name, influence, or money at my back to assist me. The A. V. C. never was supported by the powerful name which gives support to this "Subscription Plan" in Boston. The moment the A. V. C. became an organized institution, the "Subscription Plan" became a dead letter by request of the Regents of the University of New York. If, as a private individual, I was carrying it out for a worthy object, it was because I had no other means, and the school had to develop by slow growth. The Veterinary profession was scarcely born. To-day the "Subscription Plan" has no reason for existing, and has been dead for four years. I have always told you, even from the very beginning, that I was opposed to it and never liked it. So long as the question to-day has reference to Harvard, I must say there is but one way to look at it. Either Harvard has a Veterinary Department connected with it, or not; if so, it is to be regretted that so noted a University should be reduced to such an extreme as to have to advertize cheap work to recommend her educational department. If, on the other hand, the Veterinary Department is independent of the University, and is a private and personal undertaking, then why does she allow her name to be used in connection with such a degrading scheme? A. L.

